# Temporal Expressions in English and Spanish: Influence of Typology and Metaphorical Construal

**DOI:** 10.3389/fpsyg.2020.543933

**Published:** 2020-10-16

**Authors:** Javier Valenzuela, Daniel Alcaraz Carrión

**Affiliations:** Department of English Philology, University of Murcia, Murcia, Spain

**Keywords:** time, cross-linguistic, metaphor, linguistics, typology, motion, translation

## Abstract

This study investigates how typological and metaphorical construal differences may affect the use and frequency of temporal expressions in English and Spanish. More precisely, we explore whether there are any differences between English, a *satellite-framed* language, and Spanish, a *verb-framed* language, in the use of certain temporal linguistic expressions that include a spatial, deictic component (Deictic Time), a purely temporal relation between two events (Sequential Time) or the expression of the duration of an event (Duration). To achieve this, we perform two different types of studies. First, we conduct an informational gain or loss analysis of 1,650 of English-to-Spanish translations extracted from parallel corpora. Secondly, we compare the frequency of 33 English and 27 Spanish temporal expressions in two similar written online corpora (EnTenTen and EsTenTen, respectively) and a television news spoken corpus (NewsScape). Our results suggest that English uses “deictic expressions with directional language” (explicitly stating the spatial location of the temporal event, e.g., *back in those days/in the future ahead*) much more frequently than Spanish, to the extent that such directional information is often excluded in English-to-Spanish translations. Also, sequential expressions (such as *before that*/*later than*) and duration expressions (*during the whole day*) are much more frequent in Spanish. These usage differences, explained by the variability in motion typology and metaphoric construal, open up the interesting question of how these differences in linguistic usage could affect the conceptualization of time of English and Spanish speakers.

## Introduction

English and Spanish belong to two different classes regarding the expression of motion: English is a *satellite-framed* language, expressing the path of motion in satellites (e.g., *up* and *down*) or prepositional phrases (e.g., *into/out of the house*), and including manner of motion directly in the verb (e.g., *walk*, *slide*, and *crawl*). In contrast, Spanish is a *verb-framed* language, expressing path in the main verb (e.g., *subir* ‘to go up’), and manner of motion via other grammatical means such as adjuncts (e.g., *entrar/salir corriendo* lit. ‘enter/exit running’). These distinctions have been shown to affect how people focus their attention when describing a motion scene (Slobin, 1996b, 2003): English speakers adopt a more dynamic “rhetorical style,” specifying details of manner and mentioning more complex paths, and Spanish speakers use a more static rhetorical style, describing general details of the scene and letting hearers infer the precise details about manner or path.

At the same time, time is typically conceptualized using the domain of motion (e.g., *Winter is coming*; see Bender and Beller, 2014), even in expressions that do not include spatial language, as shown, for example, by gesticulation patterns (Cienki, 1998; Casasanto and Jasmin, 2012; Pagán-Cánovas et al., 2020). Temporal information can be conveyed by locating an event in relation to the speaker (Deictic Time), in relation to another event (Sequential Time) or by expressing the duration of the event. The present study tries to examine whether the typological differences found in the domain of motion for English and Spanish will affect the usage patterns of the different types of temporal expressions in these two languages. Specifically, we want to explore the hypothesis that, in a similar way to what happens in motion, the expression of time in English will be more dynamic and will include more specific details about the temporal scene, while Spanish speakers will lean to a greater extent on the inferential work of addressees. In what follows, we will first examine more closely the typological differences in the domain of motion (see section “Typological Differences Between Languages”); in Section “Taxonomies of Time: An Overview” we will review the different types of temporal expressions in both languages and their characteristics. We will then compare both languages by looking at the informational gains or losses in translations from English into Spanish, and by examining the frequencies of the different types of temporal expressions in both languages. The paper ends with a discussion of the consequences of the differences found and suggestions for further research.

### Typological Differences Between Languages

Semantic typology examines how the different languages of the world organize, structure and express the information in our conceptual domains (Evans, 2010; Moore et al., 2015). Some well-known examples are the studies of kinship (Nerlove and Romney, 1967), color (Berlin and Kay, 1969; Kay et al., 2009), body parts (Brown, 1976; Enfield et al., 2006), or sense perception (Viberg, 1983; Enfield et al., 2006; Majid et al., 2018). But undoubtedly, the domain that has attracted the most attention is that of space (Pederson et al., 1998; Talmy, 2000a; Levinson, 2003; Levinson and Meira, 2003; Majid et al., 2004; Bohnemeyer et al., 2007). This interest is well justified, given the foundational nature of the domain of space, which acts as a cornerstone to many other domains, in a process known as metaphorical transfer (Clark, 1973; Lakoff and Johnson, 1980; Núñez and Cooperrider, 2013).

Within the spatial domain, motion has been extensively researched, especially since Talmy’s seminal work (e.g., Talmy, 1985, 1991, 2000a, b). Talmy’s proposal divides the world’s languages into two broad types depending on the way in which the different elements of a motion event are mapped onto linguistic elements: *satellite-framed languages* (henceforth S-languages) and *verb-framed* languages (V-languages).^1^ English, often cited as a prototypical example of an S-language, expresses the core component of motion, i.e., the path or trajectory of motion, in satellites (e.g., *up* and *down*) or in prepositional phrases (e.g., *into/out of the house*), while the Manner of motion is directly included in the verb (e.g., *walk*, *slide*, and *crawl*). In contrast, Spanish, a V-language, typically expresses path through the main verb (e.g., *subir* ‘to go up’), and manner of motion is expressed via other grammatical means such as adjuncts (e.g., *entrar/salir corriendo* lit. ‘enter/exit running’).

Talmy’s typological scheme has generated a great deal of research and debate in the literature on motion event descriptions. Moreover, it has sparked interest in an associated notion: the cognitive consequences that these typological distinctions could have for speakers. One of the most fruitful venues of research concerns Slobin’s work (e.g., Slobin, 1987, 1996a, 2003). Slobin examined how speakers who are about to describe a motion scene display different patterns of attention depending on their type of language. He called this process “Thinking for Speaking”: speakers attend to the aspects of reality which are more easily verbalized by their linguistic means. In other words, language directs one’s attention to particular aspects of experience, which are in this way included in the description.^2^ In English, for instance, it is natural to express the notion of manner of motion, since it is included in the verb and there are many manner of motion verbs to choose from. These verbs can also be readily combined with “satellites” (directional particles such as *to*, *toward* or *across*), which makes the construction of complex paths easier than in a language such a Spanish, which encodes the direction of motion in the verb and thus, must use a different verb for each path section. In this way, when describing a motion scene, the use of the strategy known as “clause-compacting” is more natural English than in Spanish. Slobin (1996b, p. 202) mentions as an example the sentence “*he tips him off over a cliff into the water*”, in which the different sections of a complex path are referred to by a series of conjoined satellites. In contrast, in Spanish, each path section of this example would have to be expressed with a different path verb, which is more costly and awkward. The result is that Spanish speakers, besides paying less attention to manner of motion, tend to avoid the construction of complex paths when describing motion scenes. Slobin thus considered that both languages differed in their *rhetorical* styles: Spanish speakers provide hearers with details about ground information with which they can construct a scene; their descriptions are thus more static than those by English speakers, whose rhetorical style includes more explicit descriptions about the dynamics of movement.

Another area in which Slobin found consequences of these differences is translation (Slobin, 1996a, 1997). When translating between English and Spanish,^3^ Slobin observed that only 51% of the original English manner information was maintained in the Spanish target text, the rest being omitted altogether (in translations from Spanish into English, 77% of the original Spanish manner information was kept). A similar pattern was detected for path: in English-to-Spanish translations, the Spanish target text kept only 76% of path descriptions, while English translations were faithful to the original Spanish text in 92% of cases, and even occasionally added some more path information.^4^ This phenomenon of informational gain or loss in the translation process has also been replicated using parallel corpora (Verkerk, 2013). Incidentally, Filipović (2007) and Filipović and Hijazo-Gascón (2018) have convincingly shown the important consequences that these differences in informational load could have in legal interpretation. See the example in (1):

(1) Spanish original: …*pero salió por la seven* (Filipović, 2007, p. 253)
  [lit, ‘but (he) exited by 7th Street]
  English interpretation: ‘The suspect ran up 7th Street’

In this example, the original statement used a path-verb (*salió*, ‘to exit’) to describe the motion event, which the interpreter decided to change for a manner verb (*run*); this information was not included in the original text, but the use of a manner verb can be seen as a way of increasing the naturalness of the target text, given their abundance and frequency of use. While in the traslation of a novel this is inconsequential, in the context of a legal proceeding, adding a manner alters the perception of the event; the inclusion of “running” could increase the suspicion of the defendant’s actions (e.g., if he was running, chances are he could be “escaping”).

#### Beyond Thinking for Speaking

Slobin’s work can be seen as a middle point in the full exploration of how these typological distinctions could possibly affect our conceptualization of motion, what is known as “linguistic relativity” (Whorf, 1956; Kay and Kempton, 1984). This is a highly controversial topic, but studies which report an effect of the language of motion on some cognitive aspect are not scarce; they include similarity judgments, memory tasks, categorization and even eye-tracking studies (e.g., Naigles and Terrazas, 1998; Finkbeiner et al., 2002; Kersten et al., 2004; Oh, 2004; Hohenstein, 2005; Pourcel, 2005; Cifuentes-Férez and Gentner, 2006; Papafragou and Selimis, 2010; Czechowska and Ewert, 2011; Filipović, 2011). Of course, studies that have found support for a universalist view (i.e., studies showing that linguistic representations do not have an effect on cognitive processing) are also abundant; a review of this opposing evidence can be found in Cardini (2010).

Additionally, other applications of this distinction are currently being explored, such as its effects on first language acquisition (Choi and Bowerman, 1991; Özçalışkan and Emerson, 2016), second language learning (Cadierno, 2017), and influence on bilingual speakers (Lewandowski and Özçalışkan, 2019).

Finally, it must be mentioned that the distinction between the path and manner components of motion has been also examined by neuroscience and it has been shown that the brain segregates these components, which have different neural substrates (e.g., Wu et al., 2008; Janzen et al., 2012; Bender et al., 2018). Kemmerer (2005) also showed that the spatial and temporal meanings of English prepositions can be independently impaired (for the effects of motion event perception on brain potentials, see Flecken et al., 2015).

In any case, the possibility that a specific area of language is connected to established routines of information processing in a given domain which are still active even in non-linguistic tasks is worth exploring. Language has already been shown to play a significant role in the structuring of a domain as fundamental as spatial cognition (e.g., Majid et al., 2004), which justifies the question: given the well-known influence of space and motion on time, could these typological distinctions of motion have an effect on temporal cognition? In order to answer the question adequately, we review in the next section the domain of time, paying special attention to its spatial bases.

### Taxonomies of Time: An Overview

*Over the past four decades scholars have converged on the idea that humans conceptualize time primarily in terms of space* (Núñez and Cooperrider, 2013, p. 220).

This statement by Núñez and Cooperrider (2013) is probably one of the most agreed upon ideas in cognitive science. Extensive literature has focused on how time is expressed in English. For example, it has been demonstrated that nearly every aspect of time can be expressed by means of spatial metaphors (i.e., Clark, 1973; Traugott, 1978; Lakoff and Johnson, 1980, 1999; Alverson, 1994; Evans, 2004, 2013; Radden, 2004; Moore, 2006, 2014). Though other metaphors have also been described (e.g., TIME IS A RESOURCE, TIME IS A CONTAINER or TIME IS A CHANGER; cf. Lakoff and Johnson, 1980), the TIME IS SPACE metaphor is widely accepted as the main mechanism for structuring time.

When using spatial metaphors to talk about time, English almost exclusively employs the sagittal axis, placing past events behind the speaker, e.g., *to look back into the past*, and future events in front of the speaker, e.g., *to look forward to a brighter future* (Moore, 2000, 2006; Evans, 2013). However, an examination of other modalities of communication reveals that time-space metaphors can be based on different spatial configurations. For instance, research on co-speech gestures has reported the recurrent use of the lateral axis when expressing temporal concepts, locating the past on the left and the future on the right (Cienki, 1998; Casasanto and Jasmin, 2012; Walker et al., 2014; Alcaraz Carrión, 2019), at least in cultures with left-to-right reading direction; in languages such as Hebrew or Arabic, this pattern is reversed (Tversky et al., 1991). This preference for the lateral axis in the gestural modality often causes speech-gesture incongruences, since English speakers often gesture to their left while saying *back in those days* (Alcaraz Carrión, 2019; Pagán-Cánovas et al., 2020). This preference for the lateral axis has also been reported in several psycholinguistic experiments in a variety of languages (Santiago et al., 2007, 2008; Weger and Pratt, 2008) as well as in cultural artifacts such as timelines (Davis, 2012; Coulson and Pagán Cánovas, 2014) and calendars (Sinha et al., 2014), and has been experimentally connected with the direction of writing (Casasanto and Bottini, 2010; Bottini et al., 2015). Very recent research (Callizo-Romero et al., 2020) has suggested than even within the sagittal axis “whether the past or the future is conceptualized as being located in front depends on temporal focus: the balance of attention paid to the past (tradition) and the future (progress)”. These authors examine a great variety of languages, including English data from Britain and South Africa (Callizo-Romero et al., 2020).

Taking all this into account, the research community has been trying to establish a taxonomy that can categorize the different temporal meanings that are conveyed through language. This has resulted in the creation of a plethora of classification systems, all of them grouped under the tag of Temporal Frames of Reference (T-FoR), a metaphorical counterpart to Spatial Frames of Reference. Each of these taxonomies, though, comes with its own terminology and distinctions, often resulting in overlaps among them. Bender and Beller (2014) offer an excellent review of many of these taxonomies, reviewing, for instance, the ego-based versus the field-based FoR proposed by Moore (2000, 2006, 2011), the reference-point metaphors employed by Núñez (Núñez and Sweetser, 2006; Núñez et al., 2006) as well as other systems that attempt to integrate reference points (Kranjec, 2006; Zinken, 2010; Kranjec and McDonough, 2011) or to include dynamic and static relations between the reference points (Tenbrink, 2011). In the end, as Bender and Beller (2014, p. 379) state, no definitive conclusion has been reached, since the different accounts conceptualize the T-FoR in relation to very different factors, such as which is the reference point, which is its orientation or how the deictic center may affect the referencing patterns.

This paper, however, does not intend to put some order in this “tangle of space and time” [as Núñez and Cooperrider’s (2013) expressively call it], but rather to investigate possible typological differences between English and Spanish in the expression of time. Thus, we will exclusively focus on the expression of three core temporal meanings in language: the expression of past and future with a deictic center (often called the “A-Series”), the expression of temporal sequence (“B-Series”) and the expression of temporal duration. We will follow the terminology proposed by Núñez and Cooperrider (2013) to classify temporal concepts in three main categories: Deictic Time (“D-Time”), Sequential Time (“S-Time”) and Duration.

#### Deictic Time or D-Time

D-Time, also known as tensed time or the A-Series (McTaggart, 1908; Clark, 1973) refers to a temporal construal in which there is a temporal entity (i.e., the event which is to be located temporally) which is referenced with respect to the deictic center (the ‘now,’ also called the ‘Ego’), which corresponds to the moment of the speaker’s utterance; the temporal entity and the Ego are connected by a path along a given spatial axis. Temporal events are thus arranged from this Ego-centered perspective, and are located in the past or the future. Within this category, we find two types of deictic expressions: deictic expressions with directional language (DDL) and deictic expressions with non-directional language (DnDL) (Casasanto and Jasmin, 2012; Casasanto, 2016; Broders, 2019).

DDL expressions explicitly mention the direction in which the temporal event is located (i.e., its axis). They place the past behind the speaker and the future in front of them (English and Spanish linguistic systems mostly the use of the sagittal axis). Example 2 shows a case of DDL expression in which the past is located behind the speaker, while Example 3 shows a case in which the future is in front of them.

(2) Back in those days, if you were rich you had 20 children (KOCE, Charlier Rose, 11-03-2013, NewsScape Library).
(3) I have a simple question for our American panel. Are America’s best days ahead of us or behind us? Who would say ahead? (FOX-News Hannity, 06-07-2012, NewsScape Library).

In the case of DnDL expressions, the direction in which the temporal event is to be found is not made explicit linguistically, though this linguistic structure still employs the Ego as the deictic center to attribute proximity or distance to the temporal event. Note that it should not be understood that these expressions lack directionality whatsoever, since the fact that they are located in relation to the ego implies that they must have some directionality. However, the specific directionality is not made *explicit* in the linguistic expression, and has to be inferred by other means. Example 4 shows a case of a DnDL expression in which the temporal event is located far from the speaker, while Example 5 shows an example in which the event is close to the speaker: in these two cases there is no mention of the axis (sagittal, lateral, or vertical) of the temporal event, as was done in DDL expressions, nor the specific spatial location of the event within one of those axes (on the left, behind, or above the speaker, for instance):

(4) Because what they are talking about here is not the distant past (MSNBC, The Rachel Maddow show, 17-04-2014, NewsScape Library).
(5) … the number one movie in America this weekend, “The Martian”. It’s the sci-fi thriller that takes place in the near future where … (KCBS Late Show with Stephen Colbert, 06-10-2015, NewsScape Library).

Both DDL and DnDL expressions are cases of static D-Time expressions. The addition of motion to either the Ego or the temporal event gives rise to another set of temporal construals, often known as Moving Ego (the Ego acquires motion while the temporal event is static) and Moving Time (the temporal event acquires motion while the Ego remains static). These two dynamic deictic temporal expressions have been thoroughly studied (Clark, 1973; Boroditsky and Ramscar, 2002; Gentner et al., 2002; Núñez et al., 2006), but in this paper we will exclusively focus on the static D-Time temporal expressions.

#### S-Time

The second most agreed-upon category of temporal reference is known as “Tenseless Time”, the B-Series (following McTaggart, 1908; Clark, 1973) or Sequential Time (S-Time) (Núñez and Cooperrider, 2013). In this case, the temporal construal is formed by a path and a number of events that are located in relation to each other. As Moore (2014, p. 65) states, S-Time “establishes a relation between Figure and Ground in terms of an orienting principle that applies to the Figure and the Ground equally but does not depend on the ego’s perspective.” S-Time is thus employed to express the anteriority or posteriority of one event with respect to another (Evans, 2013). For instance, Example 6 shows a case in which emphasis is given to the anteriority of the event, while in 7 it is given to the posteriority of the event; in both cases, temporal location is referenced to the position of one event with respect to the other, independently of their deictic position with respect to the speaker.

(6) Literally, the calm before the storm which should hit here in the next few hours and last most of the week (KNBC, KNBC 4 News at 9 pm, 04-01-2016, NewsScape Library).
(7) Ukraine expresses optimism in the peace process after multilateral talks in Paris (KCET Deutsche Welle Journal, 02-10-2015, NewsScape Library).

#### Duration

The last temporal meaning that we will be referring to in this paper is the concept of temporal duration. The concept of duration is radically different from D-Time and S-Time since, as Núñez and Cooperrider (2013) point out, duration refers to temporal *magnitude* while D-Time and S-Time refer to the *order* of a series of events. One of the ways to conceptualize Duration, or temporal extension, following Galton (2011, p. 687), could be regarded as a way of measuring the extent of separation between two points in time. This way of understanding the concept of duration exclusively relies on one dimension of space: length (Dolscheid et al., 2013). Temporal duration corresponds to the spatial extent or length in a line, and thus the duration of a temporal event can be understood by means of the space existing between two points in a line (Example 8) From now on, we will refer to expressions that refer to the temporal duration of an event by demarcating two points in time as temporal demarcative expressions.

(8) This case was handled perfectly from beginning to end. It’s unfortunate that the prosecuting … (AlJazeera, AlJazeera News, 23-10-2013, NewsScape Library).

However, temporal duration can also be expressed through a different use of spatial metaphors. Languages such as Spanish and Greek have been reported to refer to temporal duration by means of size or quantity metaphors (Casasanto et al., 2004; Casasanto, 2005, 2008, 2010; Dolscheid et al., 2013; Baksteen, 2016). The TIME IS QUANTITY metaphor has been examined by just a handful of psycholinguistic studies (Casasanto, 2010; Baksteen, 2016; Bylund and Athanasopoulos, 2017), and it is often neglected by time conceptualization scholars, who focus on the more common TIME IS SPACE construal in English.

When referring to temporal duration in terms of quantity, we no longer talk about duration in terms of the separation between two points in time (1-dimensional space), but rather as a unit located in a 3-dimensional space (Dolscheid et al., 2013). We can, for instance, *work the whole day*, *sleep through half the meeting* or *watch a bit of the film*. From now on, we will refer to these expressions as quantity temporal expressions (Example 9).

(9) This is a huge day, obviously cyber Monday is during the entire holiday season, so it’s important … (CNN, CNN Newsroom, 01-12-2014, NewsScape Library).

Research on the domain of temporal duration is scarcer than other types of temporal meanings, and, to the best of our knowledge, there are no in-depth linguistic studies that have investigated the link between the TIME IS QUANTITY metaphor and its role in the conceptualization of temporal duration. In English, for example, we can find cases in which the duration of a similar temporal event can be conceptualized through the use demarcative temporal expressions that use 1-dimensional space or quantity temporal expressions that employ quantity duration metaphors (3-dimensional space). Consider the following examples:

(10) It’s just a beautiful film from start to finish. It’s the way they wrote their villain … (CNN, CNN The eighties, 15-12-2019, NewsScape Library).
(11) … so paranoid that someone’s gonna steal the film that they have a guy that stands right behind you like this during the whole film (KNBC, Tonight Show with Jay Leno, 30-10-2008, NewsScape Library).

**FIGURE 1:**
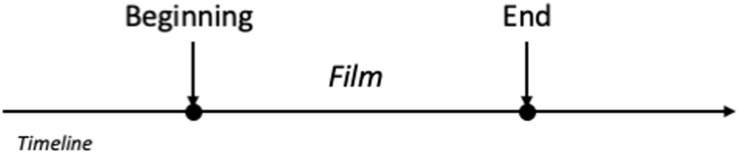
Mental representation of *from beginning.*

In Example 10, the duration of the temporal event, the film, is expressed by pinpointing the moments at which the event started and finished. This is made explicit by the demarcative temporal linguistic structure *from start to finish*, which is often accompanied by a co-speech gesture that signals two sections of a timeline (Steen et al., 2018; Valenzuela et al., 2020). The mental representation of this temporal structure might resemble Figure 1.

Example 11, however, shows a different conceptualization of the temporal event through a quantity linguistic expression. This time, the duration is no longer expressed as the 1-dimensional length between two points that signal the start and the end, but rather it is presented as a unique, integral unit. The mental representation would thus be closer to a container schema, in which the temporal event FILM is conceptualized as a unit.

**FIGURE 2:**
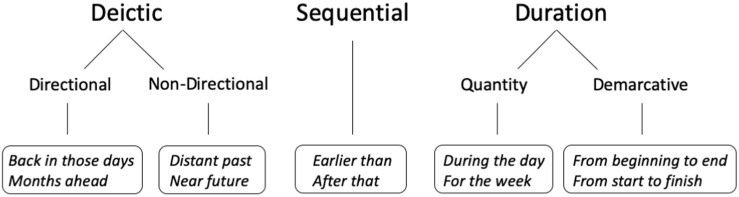
Typology of temporal events selected for our study.

In summary, we will distinguish between three main categories to express temporal information: D-Time, S-Time and Duration. D-Time expressions can be subdivided into DDL expressions, which explicitly state the direction of time, and DnDL expressions, which do not contain directional information. Lastly, duration expressions can be divided into quantity expressions, when the linguistic structure refers to time as an integral unit in a 3-dimensional space, or demarcative time expressions, when it expresses temporal duration as the 1-dimensional length between two points (see Figure 2 for a summary).

The aim of this paper is to carry out the first comparative corpus study between English and Spanish temporal expressions. We will investigate whether the typological differences between these two languages and their associated rhetorical styles might have an influence on the frequencies of deictic with directional or non-directional language, sequential and durational (quantity and demarcative) temporal expressions. We will perform a number of binary comparisons on the most frequent expressions of each of the categories in a written and an oral corpus, and will analyze the translations from English to Spanish of some of the most frequent expressions.

## Methodology

### Materials

This study employs two different corpus software programs as well as two corpora for performing the English linguistic searches. The first program used is Sketch Engine, a specialized tool for corpus research (Kilgarriff et al., 2014) which allows highly detailed corpus linguistic searches. Through this tool we have access to the English Web Corpus 2015 (EnTenTen), a corpus of 15 billion words derived from internet text. The Sketch Engine software ensures the validity of this corpus by applying deduplication tools to remove duplicated content found on the internet (including those copies that are slightly adapted from webpage to webpage). This corpus also applies the JusText tool to remove unwanted textual content which includes texts with little linguistic value, such as texts made up of incomplete sentences, advertisements, navigation menus and text snippets.

The second corpus tool we employ is CQPWeb (Hardie, 2012), a free, more flexible corpus program adapted by Peter Uhrig (Uhrig, 2018) to store the textual data from the NewsScape Television archive, a digital collection of more than 350,000 television news programs, with more than 250,000 hours of television with their associated subtitles. The result is a textual database of more than 2 billion words coming from subtitled television programs in English that have been recorded since 2004 until the present day, including channels such as CNBC, KABC, BBC, and FoxNews. This database includes television news and all types of talk shows, excluding series, films and other highly scripted television content.

The searches in Spanish were performed with the same two corpus software programs as well as the Spanish equivalents of the two English corpora. Through Sketch Engine, we used the EsTenTen2018 corpus, which belongs to the TenTen group of internet texts compiled through Sketch Engine. This Spanish web corpus contains over 17.5 billion words, including European (49%) and American (46%) Spanish, the remaining 5% being unidentified Spanish. It applies similar deduplication tools to EnTenTen 2015, as well as the JusText software to avoid the inclusion of non-relevant linguistic items. The second corpus we use is the Spanish version of the CQPWeb NewsScape Television archive corpus. Even though the content and structure of this corpus is similar to the English version, also containing television news and talk shows in both European and American Spanish, the size of the corpus is smaller (78 million words) in comparison to the English equivalent (2 billion words).

The last tool we used for this study is the online dictionary Glosbe,^5^ which allows users access to both open-source translation memories and free databases of parallel texts. The translation memories are obtained from published free parallel corpora, such as MultiUn, UN-2, EurLex-2, Europarl8, and Opensubtitles2011. Currently, the online platform offers parallel translation from English to Spanish of more than 98 million sentences. This software displays parallel translations of the original and the translated texts of the languages chosen by the researchers, allowing for the direct comparison of both excerpts.

### Linguistic Expression Selection

The search of the linguistic items is based on the five main categories that we introduced in Section “Taxonomies of Time: An Overview”: DDL, DnDL, S-Time, Demarcative, and Quantity. For each of the categories, we have elaborated a list of linguistic structures in both English and Spanish that contain keywords that express the temporal meaning of each group; we have tried to select examples which regularly show up in the literature on temporal expressions (see section “Taxonomies of Time: An Overview”). After performing the searches of all the linguistic items, we ordered them in terms of frequency for the corpus frequency comparison and chose the two most frequent expressions for each category, which were later employed in the translation analysis. The data obtained through this corpus search was employed for the informational gain or loss analysis in translation (see section “Translation Informational Gain or Loss Analysis” and “Translation”), by selecting the two most frequent expressions in English, as well as the corpus frequency comparison (see “Corpus Analysis”).

#### Deictic Expressions With Directional Language

These expressions explicitly state the direction of time in relation to a deictic center, which in English is typically the sagittal axis, locating the past behind the speaker and the future in front of them. We use as keywords the prepositions that are most commonly used in these types of expressions, with words such as *ahead*, *back*, *behind* or *in front of* (Clark, 1973; Núñez and Sweetser, 2006; Casasanto and Jasmin, 2012). To ensure that the linguistic structures always convey a temporal meaning, we search for expressions in which the prepositions are preceded or followed by a Unit of Time (UTime), which contains one of the following keywords: *hour*, *day*, *week*, *month*, *year*, and *period*. The final list of linguistic structures included in this category are the following: (*UTime*) *ahead*, *back then*, *in the* (*UTime*) *ahead*, (*UTime*) *behind*, (*Utime*) *in front of* and *back in that/those* (*UTime*). The most frequent expressions were (*UTime*) *ahead* and *back then* (see Appendix 1 for the full list of searches).

In the case of Spanish, it was complicated to find linguistic expressions which contained the full vectorial information (formed by the deictic center and a word indicating the directionality of the vector). Since there is a lack of research devoted to DDL temporal expressions in Spanish, we had to rely on our knowledge as native Spanish speakers. We searched for expressions containing the words *adelante* (lit ‘in front of’) and *detrás* (‘behind’, ‘back’), forming the combinations (*UTime*) *por delante/detrás*, such as *tenemos meses por delante* (‘we’ve got months in front of us’). We also searched the (*UTime*) *delante de* as well as (*UTime*) *detrás de*, but this resulted in a very high number of non-temporal meanings [e.g., *el conductor paró a plena luz del día delante del policía*; translated as (lit.) ‘the driver stopped in the full light of the morning in front of the police]. As a result, the most frequent expressions in this category were (*UTime*) *por delante* and (*Utime*) *por detrás* (see Appendix 2 for the full search of Spanish expressions).

#### Deictic Expressions With Non-directional Language

DnDL expressions include a deictic center but, instead of providing explicit information about the direction in which the temporal event is to be located, they just state the distance of the temporal event from the Ego; we have thus chosen keywords such as *near*, *far*, *close*, or *distant* (the same ones used in Casasanto and Jasmin, 2012). The full list of linguistic searches included: *near future*, *distant future*, *far in the future*, *close to the future*, *close in the future*, *away in the future*, *distant past*, *far in the past*, *near past*, *close to the past* and *close in the past*. In this case, we selected the three most common expressions to include both past-related and future-related structures, with *near future*, *distant future* and *distant past* being the most frequent expressions (Appendix 1).

DnDL expressions in Spanish were equivalent to the English searches, with the words *pasado* (‘past’) and *futuro* (‘future’), combined with distance adjectives (*remoto* ‘remote,’ *distante* ‘distant,’ *cercano* ‘near,’ *lejano* ‘far,’ *reciente* ‘recent,’ or *próximo* ‘near’). In this case, the most frequent expressions were *futuro cercano*, *futuro próximo*, and *pasado reciente* (see Appendix 2).

#### Sequential

This category includes expressions that describe a succession of temporal events and locate both events with respect to each other, often employing the prepositions *before* and *after* (Casasanto and Jasmin, 2012; Núñez and Cooperrider, 2013). The search items included in this category are: *previously*, *subsequently*, *after that*, *later than*, *before than* and *earlier than*. The two most frequent expressions were *previously* and *subsequently* (Appendix 1).

For S-Time expressions in Spanish, there are several equivalent phrases to the English expressions *before/after/earlier/later*, as well as *previously* and *subsequently*, namely *después de antes de*, *posteriormente/con posterioridad*, and *anteriormente/con anterioridad*. We also included other expressions that establish an anteriority/posteriority relation, such as (*Utime*) *siguiente/anterior* (*the next/previous Utime*). The most frequent expressions were *después de* and *antes de* (see Appendix 2).

#### Demarcative

Demarcative expressions express temporal duration by indicating the time-span that took place within two points in time. The [*from* X *to* Y] structure is often used to express the beginning and the end of temporal events (Steen et al., 2018). The temporal structures that were searched in the corpora are: *from start to finish*, *from beginning to end*, *from start to end*, *from genesis to revelation*, *from inception to completion* and less idiomatic cases, such as *from beginning to finish*. The two most common temporal structures were *from start to finish* and *from beginning to end* (Appendix 1).

Demarcative expressions in Spanish also have an equivalent to the English [*from* X *to* Y] construction, with the [*de/desde* X *a/hasta* Y] construction fulfilling a similar role, although the range of lexical items was more limited. The phrases that we searched were the following: *de/desde* (*el*) *principio/comienzo a/hasta* (*el*) *fin/final*. The most frequent expression was *de principio a fin*, followed by *desde el principio hasta el final* (see Appendix 2).

#### Quantity

The last category searched in this study involved Quantity expressions, which refer to duration by conceptualizing the event as a single unit. In this case, we ensured that the expression referred to duration as a unit with the inclusion of the quantity keywords *whole* and *entire*. Initially, we searched for the *for/during the whole/entire* construction, but this resulted in a high number of instances with a non-temporal meaning (e.g., *for the whole family*). We analyzed the first 100 searches and we found that only 20 cases of *for the whole* had temporal meaning; likewise, only 33 cases of *for the entire* were temporal. Thus, we decided to look for these constructions followed by a UTime (see Linguistic Expression Selection) to ensure their temporal meaning. The final list of expressions searched in this category was the following: *for the entire* (*UTime*), *for the whole* (*UTime*), *during the whole* (*UTime*) and *during the entire* (*UTime*), with *for the entire/whole* (*UTime*) being the most frequent expressions (Appendix 1).

Finally, most Quantity expressions in Spanish are also equivalent to the English linguistic expressions. In this case, we looked for linguistic expressions that followed the *durante todo/a* [DET] (*UTime*) structure (e.g., *durante todo el día* ‘during the whole day’) as well as the *todo* [DET] (*UTime*) phrase (e.g., *todo el día* ‘the whole day.’ Additionally, we also searched for variations of the same structure with the adjetives *entero* and *completo*: [DET] (*Utime*) *completo/a* and [DET] (*Utime*) *entero/a*. The most frequent expression for this category was *todo*(*s*)*/a*(*s*) *el/la/los/las* (*UTime*), followed by *durante todo*(*s*)*/a*(*s*) *el/la/los/las* (*UTime*) (see Appendix 2).

### Translation Informational Gain or Loss Analysis

We performed an informational gain or loss analysis using parallel corpora of English-to-Spanish translations (Verkerk, 2013).

For each of the two most frequent linguistic structures in the five categories, we analyzed 150 instances of the translations found in the English-to-Spanish translation memories of the online dictionary Glosbe. This meant a total of 300 translations per temporal category, except from one category in which we analyzed 450 cases (see below), amounting to a total of 1,650 instances of Spanish translations. Our information gain and loss analysis focused on the temporal component of the linguistic structure. Our aim was to establish whether the Spanish equivalent of the English temporal linguistic expression maintained the same degree of spatial information or whether, on the contrary, there were changes in the explicit spatial information conveyed by the Spanish equivalent. To this purpose, we ordered the five temporal categories (DDL, DnDL, Seq, Dem, and Quant.) in a cline in terms of the degree of explicitness of information. The most explicit pole corresponds to DDL expressions: these expressions include both a deictic center (typically, the moment of utterance) with respect to which the distance of the temporal event is located, as well as an explicit mention of the spatial axis which is activated (in English and Spanish, the most common possibility is the sagittal axis) along with the directionality within the axis (i.e., its vector), e.g., *in the months ahead*.

In DnDL, reference to the axis disappears, and we are left with relative distance (e.g., *remote past*), which is still calculated with respect to a deictic origo. Furthermore, within DnDL there are two possibilities: the inclusion of motion or its omission. In Motion-including DnDL, one of the points of the source-path-goal schema is included, either the source or the goal, but only one of them. So, for example, in expressions containing the verb *come*, we know that the goal of the motion is the deictic origo, but the source could be located in any point of 3-dimensional space (something can be coming from behind, the front, up, down, left or right). DnDL expressions which do not include the motion element just state the distance of a temporal event with respect to the deictic origo, making no reference to any axis. In *distant future*, we know that the temporal point, the future, is far from the deictic center, but we are not informed about its location (it could be in any axis) and no motion is included either toward or away from the deictic origo.

In sequential expressions, the viewpoint changes, and two temporal events are located with respect to each other, by temporal succession, instead of being exclusively based on a spatial deictic center. In these cases, a relation of posteriority and anteriority is established between two (or more) temporal events, but no reference is made to any deictic center or spatial properties such as axial location or directionality in the temporal expression itself; both temporal events can be located in the past or in the future with respect to the utterance (e.g., *after that*).

**FIGURE 3:**
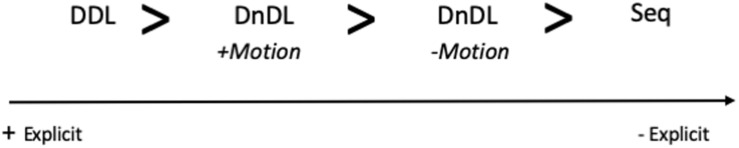
A cline of explicitness of information.

We can therefore order these types of expressions in a cline in terms of explicitness of spatial information (Figure 3).

## Results

### Translation

#### Deictic Expressions With Directional Language (DDL)

As stated in the methodology section, we searched for the two most frequent expressions for temporal deictic directional expressions in English and noted down the translations found in the parallel corpus Glosbe. The first one was (*UTime*) *ahead*. The whole list of translations is available in Appendix 3; the most common ones are listed in Table 1.

**TABLE 1:** Most frequent translations for (*UTime*) *ahead*.

Translation	# Times	Frequency	Type
(UTime) próxima/o (s)	67	44.67	DnDL(−m)
(UTime) antes	19	12.67	Sequential
(UTime) venidero(s)	14	9.33	DnDL (+m)
(UTime) siguiente	13	8.67	Sequential
(UTime) de antelación	9	6.00	Sequential
(UTime) por delante	6	4.00	DDL
(UTime) por venir	6	4.00	DnDL (+m)

We also looked for the phrase *back then*, which is the most frequent one as stated in our methodology section. There were many possibilities for translation (Appendix 3); the most common can be seen in Table 2.

**TABLE 2:** Most frequent translations for *back then*.

Translation	# Times	Frequency	Type
entonces	29	19.33	Sequential/DnDL
en/por aquel entonces	28	18.67	DnDL (−m)
en ese entonces	24	16.00	DnDL (−m)
(empty)	21	14.00	Empty
en esa época	8	5.33	DnDL (−m)
en ese momento	6	4.00	DnDL (−m)

Summarizing both tables, we see that in a vast majority of cases (more than 90%), some information is lost when translating English deictic expressions with directional language into Spanish. It must be said that in most of the cases, this loss involves the axis and its directionality, in 51% of cases the equivalent of a DDL expression was a DnDL (−m) expression: e.g., for *back then*, we would find the Spanish word *en ese momento* (lit. ‘in that moment) as an equivalent. The second most frequent strategy was eliminating the directionality, as well as the axis and the deictic point, that is, transforming the DDL expression into a S-Time one, e.g., translating *back then* for *antes* (lit. ‘before’). Table 3 offers a summary of the translation strategies.

**TABLE 3:** Translations of Deictic expressions with directional language.

English	Spanish	Total
	DnDL (−m)	51’8%
	Sequential	28,8%
	DnDL (+m)	7,6%
DDL>	DDL	3%
	Empty	7%
	Other	1.4%
	Durative	0.4%

#### Deictic Expressions With Non-directional Language (DnDL)

We searched for translations of *distant past* and *distant future*; in 98% of the cases examined, the same level of information was kept (the full list of translations can be found in Appendix 3), that is, the Spanish equivalent in the translation belonged to the same deictic non-directional type, with the same amount of information.

#### S-Time

We searched for translations of *subsequently* and *previously*; as can be seen in Appendix 3, the information tends to be kept in these. Thus, 85.1% of translations kept the same information (that is, an S-Time expression was translated by another expression belonging to the sequential type). The second more frequent strategy, which amounted to a meager 8%, involved eliminating this information altogether. Consider the following example and its translation in Spanish:

(12) English original: *The committee of Experts [*…*] has previously indicated to the State party that the employment of children constitutes dangerous work*.

Spanish translation*: La comisión de Expertos [*…*] ha señalado que el empleo de niños constituye un trabajo peligroso*.

The sequential temporal expression *previously* is removed in the Spanish translation, which only keeps the temporal marker provided by the use of the present perfect. Finally, in some cases, we did find some equivalents that opted for expressing sequential information using a deictic-non-directional; for example, translating *subsequently* for *futuro* (‘future’) or *previously* for *hasta ahora* (‘until now’).

#### Demarcative

We searched for translations of *from beginning to end* and *from start to finish*; in 98.2% of cases, the equivalents were also demarcative phrases, in which both start and end points of the temporal stretch were indicated by different lexical items, all of them roughly equivalent to ‘start’ and ‘finish’ (the full list of translations can be found in Appendix 3).

#### Quantity

Finally, we searched for the translation of the phrases *for the entire/whole* (*UTime*) (e.g., *for the entire/whole day/month/year*). The information again was kept, and a functionally equivalent option was found in virtually all cases. All the translations are available in Appendix 3.

Table 4 is a summary of the main strategies for all the translations.

**TABLE 4:** Summary of the main English-to-Spanish translation strategies.

English	Spanish	Percentage
DDL	DnDL (−m)	51’8.0%
DDL	Sequential	28’8.0%
DnDL	DnDL	96.0%
Sequential	Sequential	85.1%
Demarcative	Demarcative	98.6%
Durative	Durative	99.0%

### Corpus Analysis

#### Deictic Expressions With Directional Language (DDL)

In the case of English, the most frequent expression was the construction (*UTime*) *ahead*, which turned out to be more frequent overall in the EnTenTen corpus than in NewsScape (but was also the most frequent one in this oral corpus). The second most frequent was *back then*, with a sharp drop in the rest of options [(*UTime*) *behind/in front of*]. Combined, all these expressions rendered per-million-word (PMW) frequencies of 13.17 in EnTenTen and 31.5 in NewsScape (suggesting that this type of expression could be more natural and frequent in oral speech).

In contrast, the Spanish searches were much lower in both corpora: 0.60 PMW in EsTenTen, and 1.75 PMW in NewsScape. The full results are listed in Table 5.

**TABLE 5:** Corpus searches of DDL expressions in English and Spanish.

	EnTenTen/EsTenTen		NewsScape	
	Hits	PMW	Hits	PMW
**English**				
*(UTime) ahead back then in the (UTime) ahead (Utime) behind (Utime) in front of back in that/those (UTime)*	100,401	5.46	27,190	12.66
	84,156	4.48	22,975	10.7
	29,580	1.61	6,827	3.18
	23,468	1.28	9,336	4.35
	3,899	0.21	748	0.35
	2,344	0.13	569	0.26
Total	243,288	13.17	67,645	31.5
**Spanish**				
*(UTime)* + *por delante*	11,353	0.56	133	1.7
*(UTime)* + *por detrás*	908	0.04	4	0.05
Total	12,261	0.60	137	1.75

#### Deictic Expressions With Non-directional Language (DnDL)

In this category, the most frequent expressions in English was *near future*, with a frequency of 9.14 PMW in EnTenTen and 2.52 in NewsScape. The second and third most frequent expressions were *distant future* (0.69 PMW in EnTenTen and 0.38 in NewsScape) and *distant past* (0.47 PMW in EnTenTen and 0.12 in NewsScape; see Table 6).

**TABLE 6:** DnDL corpus searches in English and Spanish.

	EnTenTen/EsTenTen		NewsScape	
	Hits	PMW	Hits	PMW
**English**				
*Near future*	168,092	9.14	5,139	2.52
*Distant future*	12,717	0.69	823	0.38
*Distant past*	8,711	0.47	264	0.12
*Far in the future*	1,558	0.08	121	0.06
*Far in the past*	474	0.03	107	0.05
*Near past*	598	0.03	18	0.01
*Close to the future*	115	0.01	4	0
*Close to the past*	36	0.01	4	0
*Close in the future*	67	0.01	7	0
*Close in the past*	246	0.01	23	0.01
*Away in the future*	164	0.01	11	0.01
Total	192,778	10.48	6,521	3.16
**Spanish**				
*Futuro próximo*	34,661	1.71	46	0.58
*Futuro cercano*	33,698	1.66	97	1.24
*Pasado reciente*	16,982	0.84	17	0.21
*Futuro lejano*	4,054	0.2	11	0.14
*Pasado remoto*	3,491	0.17	3	0.03
*Pasado lejano*	2,207	0.11	1	0.01
*Pasado cercano*	1,429	0.07	3	0.03
*Pasado distante*	730	0.04	0	0
*Futuro distante*	664	0.03	0	0
*Pasado próximo*	399	0.02	0	0
*Futuro remoto*	375	0.02	0	0
*Futuro reciente*	101	0.01	0	0
Total	98,791	4.88	178	2.24

In Spanish, the most frequent equivalent terms were *futuro próximo* and *futuro cercano*, with a combined frequency of 3.37 PMW in EsTenTen and 1.82 PMW in NewsScape, followed by *pasado reciente* (0.84 PMW in EsTenTen and 0.21 PMW in NewsScape), showing again a lower frequency than in their English counterparts. It should be noted that in both cases, there is a lower frequency in the oral corpus.

#### S-Time

The combined frequency of the English expressions reached 117.18 PMW in SketchEngine and 74.25 PMW in NewsScape, with the most frequent expressions being *previously* and *subsequently*, as shown in Table 7 below.

**TABLE 7:** S-Time corpus searches in English and Spanish.

	EnTenTen/EsTenTen		NewsScape	
	Hits	PMW	Hits	PMW
**English**				
*Previously*	1,227,698	66.76	30,034	13.99
*Subsequently*	353,658	19.23	4,152	1.93
*After that*	301,737	16.41	84,122	39.17
*Later than*	109,258	5.94	2,675	1.25
*Before that*	104,545	5.69	32,326	15.05
*Earlier than*	57,861	3.15	6,134	2.86
Total	2,154,757	117.18	159,443	74.25
**Spanish**				
*Después de*	6,723,128	331.08	13,648	174.97
*Antes de*	5,713,152	281.34	11,677	149.7
*(UTime) siguiente*	713,128	35.12	1,347	17.26
*(UTime) anterior*	4,895,631	241.09	3,323	69.22
*Con posterioridad*	115,501	5.69	12	0.15
*Con anterioridad*	235,423	11.59	147	42.6
*Anteriormente*	1,000,338	49.26	952	12.2
*Previamente*	675,840	33.28	604	7.74
Total	19,463,885	988.45	31,710	473.84

In Spanish, the numbers are this time inverted, with a combined frequency of 988.45 PMW in EsTenTen and 473.84 PMW in NewsScape, both numbers being much higher than their English counterparts. In terms of the most frequent expressions, *después de* and *antes de* are overwhelmingly the most frequent ones, as shown in Table 8.

**TABLE 8:** Demarcative corpus searches in English and Spanish.

	EnTenTen/EsTenTen		NewsScape	
	Hits	PMW	Hits	PMW
**English**				
*From start to finish*	31,938	1.74	3,676	1.71
*From beginning to end*	13,891	0.76	1,025	0.48
*From start to end*	1,845	0.1	39	0.02
*From genesis to revelation*	1,508	0.08	9	0
*From inception to completion*	748	0.04	4	0
*From beginning to finish*	78	0.01	2	0
Total	50,008	2.73	8,429	2.21
**Spanish**				
*De principio a fin*	50,425	2.48	165	2.11
*Desde el principio hasta el final*	4,953	0.24	19	0.24
*Desde el principio hasta el fin*	1,309	0.06	4	0.05
*Desde el comienzo hasta el fin*	259	0.01	1	0.01
Total	56,946	2.79	189	2.41

#### Demarcative

There are two very frequent expressions in English, *from start to finish* and *from beginning to end*; the third more frequent is *from start to end* and there is a drop at that point with more scarce expressions, such as *from genesis to revelation*, *from inception to completion* or the very infrequent *from beginning to finish*. The frequency of these expressions is roughly the same in EnTenTen and NewsScape, as shown in Table 9.

**TABLE 9:** Quantity corpus searches in English and Spanish.

	EnTenTen/EsTenTen		NewsScape	
	Hits	PMW	Hits	PMW
**English**				
*For the entire (UTime/period)*	11,935	0.65	1,753	0.82
*For the whole (UTime/period)*	11,152	0.61	1,632	0.76
*During the whole (UTime/period)*	2,975	0.16	39	0.02
*During the entire (Utime/period)*	2,650	0.14	78	0.04
Total	26,327	1.56	338,617	1.64
**Spanish**				
*Durante todo/a(s)* + *det* + *UTime*	196,992	9.7	1,039	13.28
*Todo el (UTime)*	1,069,579	52.67	5,676	72.76
*(Det) (Utime) completo/a*	44,131	2.17	17	0.21
*(Det) (UTime) entero/a*	45,600	2.5	39	0.5
Total	1,311,158	67.04	6,771	86.75

As regards Spanish, the main demarcative phrases are formed by the combination of *de/desde principio/comienzo a/hasta el fin/final*; the combined frequencies of these phrases are found in Table 9 again.

However, it should be noted that the construction [*from* X *to* Y] is very polysemous, and tends to indicate different meanings, not all of them temporal. Thus we find spatial meanings (e.g., *from Boston to New York*), temporal meanings (e.g., *from dusk til dawn*), “inclusion” meanings (e.g., *from apples to oranges*, *all fruit is healthy*), “interval” meanings (e.g., *from 140 to 147 pounds*, *you are a Welter*). Something similar happens in Spanish, where the construction [*de* X *a* Y] covers the same range of meanings approximately. Thus, some of our results were not temporal (e.g., *from the beginning to the end of the road*). To try to quantify the number of temporal readings of our sample, we randomly generated three sets of 100 sentences, and marked temporal readings in constructions of both languages. We found that the percentage of temporal meanings in both languages was different: 81% of demarcative phrases in English were temporal, while only 63% of Spanish demarcative phrase demarcative phrases could be considered temporal. That means that the final results of the Table 9 should be modulated; applying this correction, we find a frequency of English temporal demarcatives of 2.05 PMW vs. 1.68 PMW in Spanish (in the TenTen corpus) and a frequency of 1.85 PMW in English and 1.44 PMW in Spanish in the NewsScape corpus. In both cases, the frequency was higher in English. Even though the frequency of the expressions in this category is more similar that in the rest of the categories, a Chi^2^ test suggests that the difference is highly significant (χ^2^ = 763,1422717; *p* < 0.001).

#### Quantity

Finally, we searched for expressions which make reference to the whole duration of a temporal event; in English, we chose *for/during the entire/whole* (*UTime*) (e.g., *for the whole week; during the entire day*). In Spanish, as indicated by the equivalent phrases found in the translation part, we included *durante todo/a* [DET] (*UTime*), e.g., *durante todo el día* ‘during the whole day.’ Since the translation equivalent of a durative phrase in English includes most of the time a phrase starting with *todo*, with no equivalent for *during* or *for*, we also included the phrase *todo* [DET] (*UTime*), e.g., *todo el día* ‘the whole day.’ We found that durative phrases were much more frequent in Spanish (67.04 PMW/86.75 PMW) than in English (1.56 and 1.64 PMW), as shown in Table 10.

**TABLE 10:** PMW frequencies of temporal phrase types in English and Spanish.

Type of temporal expression	EnTenTen/EsTenTen	NewsScape
English DDL	13.17	31.5
Spanish DDL	0.6	1.75
English DnDL	10.48	3.16
Spanish DnDL	4.88	2.24
English sequential	117.18	74.25
Spanish sequential	988.45	473.84
English demarcative	2.05	1.85
Spanish demarcative	1.68	1.44
English durative	1.56	1.64
Spanish durative	67.04	86.75

A summary of the differences of all the categories we have covered can be seen in Table 7.

## Discussion

Our results show a very consistent pattern in the usage differences of English and Spanish temporal expressions, which becomes apparent through the two methods we have used, which can be seen as complementing each other. First, there is a very clear loss of explicit information in the translation of Deictic expressions with directional language from English into Spanish. This fact parallels the informational losses found in the case of motion, and can initially be explained by the same mechanism. Slobin (1996b) found that elicited narratives in Spanish display fewer complex paths than English descriptions; English can construct complex paths with the concatenation of “satellites” (*clause-compacting*), which are then simplified when translated into Spanish, many details of the path being lost in the process. Spanish, as we have seen, expresses directional path preferentially with a verb, an element which is always more costly (verbs are connected to predicate-argument structures, sometimes involving several arguments). Thus, it seems that the typological make-up of English facilitates an explicit reference to the vector-based direction of timelines, in stark contrast with Spanish. It should be noted that Spanish has an abundance of verbs which conflate path-information (e.g., subir –go up, bajar –go down); however, most frequently, English temporal expressions convey the spatial information in elements which are not verbs (e.g., *I saw what happened back then*). As happens in this and many other examples the spatial information is contained in a preposition, not in the verb, and thus, the Spanish translation does not include any motion verb either. While in the translation of pure physical motion events, spatial information contained in prepositions may surface in a verb of motion in Spanish, this has not been the case in the translation of temporal expressions containing spatial information.

It could be objected that this loss of explicit information does not necessarily entail that the information is lost altogether. For example, when a DDL expression is translated to a DnDL expression in Spanish, the default situation in the Spanish equivalent would probably still evoke a sagittal timeline, with future in the front and past in the back, which is, after all, the main axis used in the Spanish linguistic system (cf. *dejamos atrás malos recuerdos*, lit. ‘we leave behind us bad memories’). However, this is not the only possibility: since the lateral timeline is also active in speech (Casasanto and Jasmin, 2012; Walker et al., 2014), with past on the left and future on the right, for a sentence such as *en aquellos lejanos momentos de mi infancia* ‘in those remote moments of my childhood,’ there are at least two logical possibilities to locate those moments: behind the speaker or to their left. Expressions such as ‘*back then*,’ which explicitly point at a sagittal point behind the speaker, are translated by *en ese momento* (‘in that moment’), which only keep the notion of distance from the speaker. The exact temporal point of where the moment is located has to be recovered from contextual cues, in a manner reminiscent of other ungrammaticalized notions such as number in Chinese or tense in Indonesian (Boroditsky et al., 2002; Sun, 2006). Moreover, another strategy when translating DDL expressions turns them into S-Time expressions, where the loss of explicitness is even greater, since not only directionality but also the deictic center as main mechanism for the location of an event is lost. This is what happens, for example, when the English phrase *back then* is translated by *antes de eso* (‘before that’). This can be connected to the increased Spanish usage of sequential expressions, as we comment below. Finally, other temporal expressions in English (DnDL, S-Time, Demarcative, and Quantity), are not as strongly linked with free lexical morphemes pertaining to space (that is, no satellites), and accordingly, do not evidence any information gain or loss of explicitness.

**FIGURE 4:**
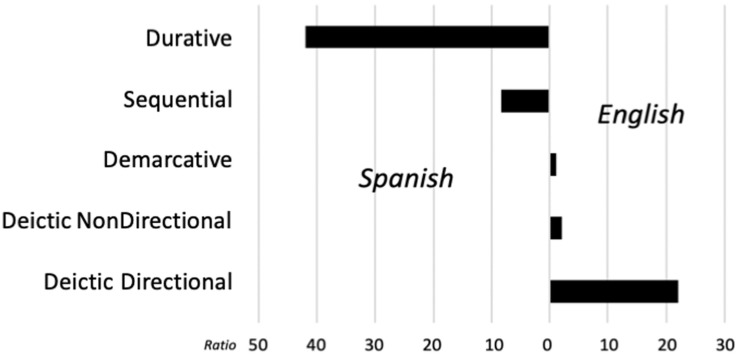
Ratios between English and Spanish of the different temporal expressions.

Regarding the second part of the study, the differences found in the frequency of expressions belonging to the different types, we found that DDL expressions are much more frequent in English than in Spanish (Figure 4). This difference could be explained by the same typological differences which explain the informational loss of path in English-to-Spanish translation; since direct and complete reference to directional information is hindered by Spanish typological make-up, these types of expressions have a much lower frequency in that language.

What is more striking, though, is the very clear differences among the other types. Quantity expressions, for example, are extremely more frequent in Spanish than in English. One possible explanation rests on the existence of the *DURATION-AS-DISTANCE* vs. *DURATION-AS-QUANTITY* metaphors identified in the literature (Casasanto, 2010; Baksteen, 2016; Bylund and Athanasopoulos, 2017). If Spanish speakers are more used to quantify time using expressions such as *mucho tiempo* (‘much time’), it makes sense that they should more easily construe durations as “wholes” than English speakers. This could also explain why temporal demarcatives, though overall less frequent than other types of temporal expressions, are also less frequent in Spanish than in English. The English phrase *from beginning to end* assumes a spatial timeline, conveying the duration of a given event by signaling the starting and end points in that spatial timeline. In contrast to this English *duration-as-distance* construal, Spanish speakers would opt for a *duration-as-quantity*, which could explain their lower use of demarcatives.

How could this combination of preferential activation of directed spatialization plus difference between *DURATION-AS-DISTANCE/AMOUNT* construals affect other categories? The explanation could come from what Slobin (1991) called “rhetorical styles”: in English, motion scenes are described dynamically, providing abundant details about paths and manner; in contrast, Spanish speakers omit these details, opting for more static descriptions, and letting hearers infer path details (as well as manner). In the same way, it could be argued that, though deictic reference is widely used in Spanish, its speakers take the same route and opt for a strategy in which the temporal location of an event is referred to another event, downplaying the role of the deictic origin to the moment of speech, and thus letting the hearer infer the exact moment in which the sequence took place from information outside the temporal expression itself. This would explain why we also find that deictic expressions with non-directional language are also more frequent in English than in Spanish. These results, though, should be taken with some reservations, since we find one specific English phrase (*distant future*) which is much more frequent than the rest, and tilts the results toward that language.

What should we make of these differences? Time conceptualization has been described as a combination of (nearly) universal features and culture-specific particularities (Bender and Beller, 2014). So, while temporal linearity seems to be extremely common across the world’s cultures (McCormack, 2015; McCormack and Hoerl, 2017), there are also very clear cultural differences in how that linearity is implemented (with different axes, directionalities, etc.). These differences become conventionalized through processes of cultural transmission (especially language; Nelson, 1996; Zhang and Hudson, 2018). As Nelson (1996) argues, “language may make salient a type of relation that was not previously apparent in the child’s non-linguistic conceptual representations.” How language builds on pre-linguistic cognitive abilities, structuring them in order to conform the adult cognitive system, is indeed a topic with a long tradition [e.g., Vygotsky, 1962; see Gentner (2003) and Christie and Gentner (2014) for the influence of language on relational concepts].

A concrete example is described in Dolscheid et al. (2014), who found that in some languages (such as English) pitches are described as “high” or “low” (height-metaphor), while in others, they are described as “thin” or “thick” (thickness-metaphor). In their study, prelinguistic infants (4 months old) were sensitive to both mappings, which made the authors conclude that “language builds on preexisting mappings, changing them gradually via competitive associative learning. Space-pitch mappings that are language-specific in adults develop from mappings that may be universal in infants” (Dolscheid et al., 2014, p. 1,256). We could speculate that a similar process could be at play in the case of temporal conceptualization. In acquisition, children have been shown to form categories of path before manner (Konishi et al., 2016; Ji and Hohenstein, 2018). At the same time, there is evidence that sequential understanding is found much earlier (even in 11-month-old infants; O’Connell and Gerard, 1985; Bauer and Mandler, 1992) than timelines, which are acquired later (Tilman et al., 2018). That means that both sequentiality and attention to path are developed first, leaving a developmental temporal span for establishing a connection between the increased attention to the paths of motion in both domains: English speakers would become used to an heightened specification of both spatial paths and explicitation of spatialized deictic location, while Spanish speakers would learn to rely on the inferential capacities of hearers in both domains.

Something similar could be happening when it comes to duration. Pre-linguistic infants are sensitive to the links between duration and spatial extent (Srinivasan and Carey, 2010), and also between duration and size (Lourenco and Longo, 2011). So, as in the case of Dolscheid et al. (2014), both *DURATION-AS-DISTANCE* and *DURATION-AS-QUANTITY* are present pre-linguistically, and one of these two possibilities would be selected by linguistic usage, accordingly re-arranging the pattern of temporal type usages seen in our study.

Hubbard and Teuscher (2010) offer a neurological explanation, pointing at some neural structures (in the parietal cortex) which “create a predisposition toward a neural mapping between the domains of time and space, and thus provide a brain-based constraint on the universal TIME IS SPACE metaphor.” In the opinion of these authors, and consistently with Dolscheid et al. (2014) proposal, “cultural artifacts that best fit the pre-existing structures of the brain are most easily learned, and are therefore most likely to be passed on to future generations.”

The present study opens up a novel method for approaching the problem of crosslinguistic temporal language: the careful analysis of the usage patterns of different types of expressions, both in their frequencies and in the differences in informational load of translational equivalents. The well-attested connections between linguistic forms and the shape of the cognitive structures we construct provide a firm theoretical support to the importance of studies like the present one. We hope to have shown that the differences in the use of different types of temporal expressions in English and Spanish are not only real (and so far, undocumented), but that these differences are not haphazard, and can be explained by typological differences in the area of motion and different construals of time in the two languages compared. This opens up new and exciting paths of research aimed at finding the potential consequences that these differences may have for the temporal conceptualization patterns of English and Spanish speakers.

## Data Availability Statement

All datasets generated for this study are included in the article/Supplementary Material.

## Author Contributions

JV and DAC contributed in the conception and design of the study and performed the corpus analysis. JV wrote the introduction to motion typology and performed the translation analysis. DAC wrote the introduction to temporal typologies and collected the data. The discussion was written by JV, with support and comments from DAC. Both authors contributed to manuscript revisions and approved the final submitted version.

## Conflict of Interest

The authors declare that the research was conducted in the absence of any commercial or financial relationships that could be construed as a potential conflict of interest.

## Footnotes

1 Since its initial proposal this dichotomy has been further elaborated and a third typological group has been proposed (*equipollently-framed* languages), corresponding to languages whose morphosyntactic constituents expressing path and manner have equal status. Even more recently, the whole system has started to be redefined in terms of clines: languages can be categorized along a “manner-salience cline” (Slobin, 2004) and a “path-salience cline” (Ibarretxe-Antuñano, 2009). Since the focus of this paper does not lie on how to best categorize the world’s languages regarding the expression of motion, focusing instead on the consequences of the typological differences between English and Spanish, which fall squarely in the satellite- and verb-framed poles of the distinction, we will not elaborate this issue further.

2 A compilation of studies of more than 90 languages can be found in Berman and Slobin (1994) and Strömqvist and Verhoeven (2004).

3 For translations from English into other languages, see Slobin (2005).

4 See Ibarretxe-Antuñano and Filipović (2013) for a list of strategies that translators apply with respect to manner, path and the motion event.

5 https://glosbe.com/
