# Supplementary material for: Temporal Expressions in English and Spanish: Influence of Typology and Metaphorical Construal
Source: Front Psychol. 2020 Oct 16;11:543933. doi: 10.3389/fpsyg.2020.543933 (PMC7607253; doi:10.3389/fpsyg.2020.543933)
Supplement: Supplementary file 1 [file Data_Sheet_1.pdf]

Appendix 1. English temporal expression corpus searches

Deictic with Directional Language (DDL)

	EnTenTen2015		NewsScape	
	**Hits**	**PMW**	**Hits**	**PMW**
(UTime) *ahead*	100,401	5.46	27,190	12.66
back then	84,156	4.48	22,975	10.7
in the (UTime) *ahead*	2,958	1.61	6,827	3.18
(Utime) *behind**	23,468	1.28	9,336	4.35
(Utime) *in front of*	3,899	0.21	748	0.35
back in that/those (UTime)	2,344	0.13	569	0.26
TOTAL	217,226	13.17	67,645	31.5

Deictic with non-directional (DnDL)

	EnTenTen2015		NewsScape	
	**Hits**	**PMW**	**Hits**	**PMW**
near future	168,092	9.14	5,139	2.52
distant future	12,717	0.69	823	0.38
distant past	8,711	0.47	264	0.12
far in the future	1,558	0.08	121	0.06
far in the past	474	0.03	107	0.05
near past	598	0.03	18	0.01
close to the future	115	0.01	4	0
close to the past	36	0.01	4	0
close in the future	67	0.01	7	0
*close in the past**	246	0.01	23	0.01
away in the future	164	0.01	11	0.01
TOTAL	192,778	10.48	6,521	3.16

Sequential

	EnTenTen2015		NewsScape	
	**Hits**	**PMW**	**Hits**	**PMW**
previously	1,227,698	66.76	30,034	13.99
subsequently	353,658	19.23	4,152	1.93
after that	301,737	16.41	84,122	39.17
later than	109,258	5.94	2,675	1.25
before that	104,545	5.69	32,326	15.05
earlier than	57,861	3.15	6,134	2.86
TOTAL	2,154,757	117.18	159,443	74.26

Demarcative

	EnTenTen2015		NewsScape	
	**Hits**	**PMW**	**Hits**	**PMW**
from start to finish	31,938	1.74	3,676	1.71
from beginning to end	13,891	0.76	1,025	0.48
from start to end	1,845	0.1	39	0.02
from genesis to revelation	1,508	0.08	9	0
from inception to completion	748	0.04	4	0
from beginning to finish	78	0.01	2	0
TOTAL	50,008	2.72	4,755	2.21

Quantity

	EnTenTen2015		NewsScape	
	**Hits**	**PMW**	**Hits**	**PMW**
for the entire (UTime/period)	11,935	0.65	1,753	0.82
for the whole (UTime/period)	11,152	0.61	1,632	0.76
during the whole (UTime/period)	2,975	0.16	39	0.02
during the entire (Utime/period)	2,650	0.14	78	0.04
TOTAL	28,712	1.56	3,502	1.64

Appendix 2. Spanish temporal expressions corpus searches

Deictic with Directional Language (DDL)

	EsTenTen2018		NewsScape	
	**Hits**	**PMW**	**Hits**	**PMW**
(UTime)+por delante	11,353	0.56	133	1.7
(UTime)+por detrás	908	0.04	4	0.05
TOTAL	12,261	0.60	137	1.75

Deictic with non-Directional Language (DnDL)

	EsTenTen2018		NewsScape	
	**Hits**	**PMW**	**Hits**	**PMW**
futuro próximo	34,661	1.71	46	0.58
futuro cercano	33,698	1.66	97	1.24
pasado reciente	16,982	0.84	17	0.21
pasado remoto	3,491	0.17	3	0.03
pasado lejano	2,207	0.11	1	0.01
pasado cercano	1,429	0.07	3	0.03
pasado distante	730	0.04	0	0
pasado próximo	399	0.02	0	0
Futuro reciente	101	0.01	0	0
TOTAL	93,698	4.63	167	2.1

Sequential

	EsTenTen2018		NewsScape	
	**Hits**	**PMW**	**Hits**	**PMW**
después de	6,723,128	331.08	13,648	174.97
antes de	5,713,152	281.34	11,677	149.7
(UTiempo) anterior	4,895,631	241.09	3,323	69.22
anteriormente	1,000,338	49.26	952	12.2
previamente	675,840	33.28	604	7.74
(UTiempo) siguiente	713	35.12	1,347	17.26
con anterioridad	235	11.59	147	42.6
con posterioridad	116	5.69	12	0.15
TOTAL	19,009,153	988.45	31,710	473.84

Demarcative

	EsTenTen2018		NewsScape	
	**Hits**	**PMW**	**Hits**	**PMW**
de principio a fin	50,425	2.48	165	2.11
desde el principio hasta el final	4,953	0.24	19	0.24
desde el principio hasta el fin	1,309	0.06	4	0.05
desde el comienzo hasta el fin	259	0.01	1	0.01
TOTAL	56,946	2.79	189	2.41

Quantity

	EsTenTen2018		NewsScape	
	**Hits**	**PMW**	**Hits**	**PMW**
durante todo/a(s) + det + UTime	196,992	9.7	1,039	13.28
todo el (UTime)	1,069,579	52.67	5,676	72.76
(Det) (Utime) completo/a	44,131	2.17	17	0.21
(Det) (UTime) entero/a	45,600	2.5	39	0.5
Total	1,311,158	67.04	6,771	86.75

Appendix 3. Translations from English to Spanish

Deictic with Directional Language (DDL)

(<u>UTime) ahead</u>

Translation	#Times	Frequency	Type
(UTime) próxima/o (s)	67	44.67	DnDL (-m)
(UTime) antes	19	12.67	Sequential
(UTime) venidero(s)	14	9.33	DnDL (+m)
(UTime) siguiente	13	8.67	Sequential
(UTime) de antelación	9	6.00	Sequential
(UTime) por delante	6	4.00	DDL
(UTime) por venir	6	4.00	DnDL (+m)[1]
(UTime) anterior	4	2.67	Sequential
(UTime) que viene	2	1.34	DnDL (+m)
(UTime) de anticipación	2	1.33	Sequential
(UTime) de adelanto	1	0.67	DDL
(UTime) de anterioridad	1	0.67	Sequential
(UTime) que comienza	1	0.67	DnDL (+m)
(UTime) futuro/a(s)	1	0.67	DnDL (-m)
(UTime) ante nosotros	1	0.67	DDL
durante largo tiempo	1	0.67	Durative
a (UTime) de	1	0.67	Other
Los (UTime) que nos esperan	1	0.67	Other

<u>Back then</u>

Translation	#Times	Frequency	Type
entonces	29	19.33	Sequential/DnDL 8-m)
en/por aquel entonces	28	18.67	DnDL (-m)
en ese entonces	24	16.00	DnDL (-m)
(empty)	21	14.00	Empty
en esa época	8	5.33	DnDL (-m)
en ese momento	6	4.00	DnDL (-m)
antes	4	2.67	Sequential
en ese tiempo	4	2.67	DnDL (-m)
en aquella época	4	2.67	DnDL (-m)
después	3	2.00	Sequential
hace tiempo	3	2.00	Other/DnDL
por entonces	2	1.33	DnDL (-m)
en aquellos días	2	1.33	DnDL (-m)
en aquel tiempo	2	1.33	DnDL (-m)

[1] +m and -m indicate “plus motion” or “minus motion”

en aquel momento	2	1.33	DnDL (-m)
antes de eso	1	0.67	Sequential
en el pasado	1	0.67	DnDL (-m)
de la época	1	0.67	Other
ya entonces	1	0.67	DnDL (-m)
luego	1	0.67	Sequential
años atrás	1	0.67	DDL
en los viejos tiempos	1	0.67	Other

Deictic with non-Directional Language (DnDL)

<u>Distant past</u>

**Translation**	**#Times**	**Frequency**	**Type**
pasado lejano	67	44.67	DnDL (-m)
pasado distante	43	28.67	DnDL (-m)
pasado remoto	32	21.33	DnDL (-m)
tiempos remotos	3	2.00	DnDL (-m)
remontar al pasado	2	1.33	DnDL (-m)
tiempo lejano	1	0.67	DnDL (-m)
tiempo atrás	1	0.67	DDL
tiempos lejanos	1	0.67	DnDL (-m)

<u>Distant future</u>

**Translation**	**#Times**	**Frequency**	**Type**
futuro lejano	107	71.33	DnDL (-m)
futuro distante	34	22.67	DnDL (-m)
a largo plazo	4	2.67	DnDL (-m)
futuro remoto	1	0.67	DnDL (-m)
futuro próximo	1	0.67	DnDL (-m)
perspectivas	1	0.67	Other
pasado cierto tiempo	1	0.67	DnDL (+m)
futuro remoto	1	0.67	DnDL (-m)

Sequential

<u>Subsequently</u>

**Translation**	**#Times**	**Frequency**	**Type**
posterior(es)	36	24.00	Sequential
posteriormente	33	22.00	Sequential
ulterior(es)	14	9.33	Sequential
(empty)	13	8.67	Empty

subsiguiente(s)	11	7.33	Sequential
después	7	4.67	Sequential
ulteriormente	6	4.00	Sequential
siguientes	6	4.00	Sequential
a continuación	3	2.00	Sequential
consiguiente	3	2.00	Sequential
consecutiva	2	1.33	Sequential
futuros	2	1.33	DnDL (-m)
luego	2	1.33	Sequential
a raíz de	2	1.33	Other
subsecuente	2	1.33	Sequential
subsiguientemente	1	0.67	Sequential
consecuente	1	0.67	Sequential
como consecuencia de	1	0.67	Sequential
en consecuencia	1	0.67	Sequential
a posteriori	1	0.67	Sequential
con posterioridad	1	0.67	Sequential
más tarde	1	0.67	Sequential
tras	1	0.67	Sequential

Previously

Translation	#Times	Frequency	Type
anteriormente	53	35.33	Sequential
previamente	39	26.00	Sequential
antes	18	12.00	Sequential
(empty)	11	7.33	Empty
anterior(es)	7	4.67	Sequential
ya	6	4.00	DnDL (-m)
con anterioridad	4	2.67	Sequential
habían sido	4	2.67	DnDL (-m)
hasta ahora	3	2.00	DnDL (-m)
hasta entonces	3	2.00	DnDL (-m)
hasta la fecha	1	0.67	DnDL (-m)
con antelación	1	0.67	Sequential

Demarcative

From start to finish

Translation	#Times	Frequency	Type
De principio a fin	84	55.63	Dem
Desde el principio hasta el final	24	15.89	Dem
Desde le principio hasta el fin	13	8.61	Dem
Desde el inicio hasta el fin	6	3.98	Dem

Desde el comienzo hasta el fin	4	2.65	Dem
Desde el comienzo hasta el final	4	2.65	Dem
todo	3	1.99	Quantity
De inicio a fin	3	1.99	Dem
De comienzo a fin	2	1.32	Dem
A placer	1	0.66	Other
Desde la salida hasta la llegada	1	0.66	Other
De cabo a rabo	1	0.66	Dem
De un extremo a otro	1	0.66	Other
Desde su inicio hasta su finalización	1	0.66	Dem
Todo el tiempo	1	0.66	Quantity
empty	1	0.66	Other

From beginning to end

**Translation**	**#Times**	**Frequency**	**Type**
de principio a fin	90	62.50	Dem
desde el principio hasta el fin	14	9.72	Dem
desde el comienzo hasta el fin	8	5.56	Dem
desde el principio hasta el final	8	5.56	Dem
desde el comienzo hasta el final	6	4.17	Dem
de comienzo a fin	5	3.47	Dem
de inicio a fin	5	3.47	Dem
desde el principio al final	4	2.78	Dem
desde el principio al fin	4	2.78	Dem
desde el comienzo al final	3	2.08	Dem
de cabo a rabo	2	1.39	Dem
desde su inicio hasta su conclusión	1	0.69	Dem

Quantity

For the entire (UTime)

**Translation**	**#Times**	**Frequency**	**Type**
todo el (UTime)	49	32.67	Quantity
durante toda/o(s) la (UTime)	32	21.33	Quantity
durante la totalidad de la (UTime)	16	10.67	Quantity
durante la (UTime)	12	8	Quantity
para/por todo el (UTime)	10	6.67	Quantity
por/para toda la (UTime)	8	5.33	Quantity
durante (UTime) enteros	6	4	Quantity
por/para la (Utime) entera	6	4	Quantity
por/para la (UTime) completa	5	3.33	Quantity
(UTime) completa	3	2	Quantity
a lo largo de todo el (UTime)	1	0.67	Space

de la (UTime)	1	0.67	Quantity
el conjunto del (UTime)	1	0.67	Quantity

For the whole (UTime)

**Translation**	**#Times**	**Frequency**	**Type**
por toda la (UTime)	47	31.33	Quantity
toda la (Utime)	38	25.33	Quantity
durante toda la (Utime)	31	20.67	Quantity
la (Utime) completa	12	8.00	Quantity
la (UTime) entera	8	5.33	Quantity
durante la (UTime)	5	3.33	Quantity
por (UTime)	4	2.67	Quantity
el (Utime) en su conjunto	2	1.33	Quantity
por/para la totalidad del (Utime)	2	1.33	Quantity
durante la (Utime) entera	1	0.67	Quantity
